# Community Awareness and Perceptions of Genitourinary Malformations: A Cross-Sectional Survey Study

**DOI:** 10.3390/healthcare12242558

**Published:** 2024-12-19

**Authors:** Ahmad A. Al Abdulqader, Haytham Mohammed Alarfaj, Mohammed Saad Bu Bshait, Ahmed Hassan Kamal, Mohammed Nasser Albarqi, Amnah Ali Alkhawajah, Alreem I. Alshahri, Abdullah Abduljalil Almubarak, Mariyyah Abdullah Almuhaini, Nawaf Al Khashram, Abdullah Almaqhawi, Ossama Mohamed Zakaria

**Affiliations:** 1 Departments of Surgery, College of Medicine, King Faisal University, Al Hofuf P.O. Box 400, Saudi Arabia; a.alabdulqader@yahoo.com (A.A.A.A.); haytham_alarfaj@hotmail.com (H.M.A.); mbubshait@kfu.edu.sa (M.S.B.B.); dr.ahmedhk81@gmail.com (A.H.K.); ossamaz2004@gmail.com (O.M.Z.); 2 Department of Family and Community Medicine, College of Medicine, King Faisal University, Al Hofuf P.O. Box 400, Saudi Arabia; dr.anma@hotmail.com; 3 College of Medicine, King Faisal University, Al Hofuf P.O. Box 400, Saudi Arabia; amnahalkhawajah@gmail.com (A.A.A.); alreemibrahim778@gmail.com (A.I.A.); abdullahjhm@gmail.com (A.A.A.); mariyyaham@gmail.com (M.A.A.); 4 Department of Biomedical Sciences, College of Medicine, King Faisal University, Al Hofuf P.O. Box 400, Saudi Arabia; nalkhashram@kfu.edu.sa

**Keywords:** genitourinary malformations, risk factors, knowledge, community perception

## Abstract

Background and Objectives: On a local and national scale, genitourinary malformations (GUMs) are the second most encountered congenital anomaly in children. GUMs are linked to several risk factors, including maternal co-morbidities and insufficient folic acid. They may also be related to maternal health and care during pregnancy. Expanding our knowledge about these factors is necessary for the development of preventative measures, which could reduce GUM incidence. This study evaluated the local youth’s understanding and perceptions of genitourinary anomalies. Materials and Methods: This cross-sectional, qualitative, anonymous, questionnaire-based study involved members of the local population, aged 18 years or over. Based on a 5% type I error rate (α = 0.05) and an 80% response rate, a sample size of 481 was determined. The questionnaire was completed by 902 people. The data were analyzed using SPSS version 25 (IBM). Results: Over half (57%) of respondents believed that hormonal therapy during pregnancy could increase GUM risk. Moreover, 46% thought that maternal chronic diseases could be another risk factor, while 43% believed that pregnancy-related conditions, such as pre-eclampsia, increased GUM risk. Women had higher odds of high perception scores than men, according to the univariate and multivariate analyses. Most participants (74%) strongly agreed that proper and ongoing prenatal follow-ups are necessary, 69% agreed that premarital medical check-ups are necessary, and 67% believed that optimal nutrition throughout pregnancy is necessary to reduce GUM risk. Conclusions: The results emphasize the necessity of developing healthcare strategies specifically designed to increase knowledge about GUMs and overcome incorrect community perceptions of risk factors that could also help improve attitudes towards prevention and ultimately reduce the incidence of GUMs.

## 1. Introduction

Genitourinary malformations (GUMs) are the most common congenital anomalies in children. Their overall incidence ranges from 1 to 4 per 1000 pregnancies [1,2]. Yet, on a local scale, congenital anomalies are reported in 41 out of every 1000 births. Among these, GUMs are the second most frequent birth anomalies (11.3/1000) [3,4].

GUMs may predispose the patients to serious morbidities, such as psychosocial difficulties, sexual dysfunction, infertility, and impaired renal function [5,6]. Therefore, prenatal evaluation and early identification are essential to mitigate such consequences [7,8].

Despite efforts to improve prenatal screening and detection, preventative measures and structured educational programs are still not widely implemented [3]. Many studies have highlighted the inadequacy of public knowledge as regards congenital malformations and their related causes. Furthermore, incorrect beliefs and notions about the etiology of congenital abnormalities exist within certain communities. Overall, accurate knowledge about risk factors is limited [8,9]. Moreover, numerous studies highlight that awareness initiatives aimed at healthcare providers and families can improve comprehension of congenital anomalies and the significance of preconception care. Educating healthcare workers on the importance of screening, early diagnosis, and family counseling can enhance the management of congenital anomalies [10,11].

Several risk factors have been linked to the incidence of GUMs. Some of these risk factors are preventable or modifiable, such as folic acid deficiency, radiation exposure, consanguinity, advanced maternal age, and maternal co-morbidities [3]. Thus, comprehensive information and knowledge about such risk factors is necessary in order to develop appropriate preventative measures that could reduce the incidence of GUMs. This study aims to assess community knowledge, perceptions, and attitudes regarding GUMs to identify gaps in awareness and potential areas for targeted educational interventions.

## 2. Materials and Methods

### 2.1. Study Design and Setting

A local community-based, descriptive, cross-sectional study was conducted. All local adult residents who agreed to participate were included. Assuming a predetermined acceptable margin of error of 5% (d = 0.05) and that 50% of the population was familiar with GUMs, the minimum sample size was calculated to be 385 for a 95% confidence interval. Assuming a type I error rate of 5% (α = 0.05) and an 80% response rate, the required sample size was determined to be 481. Nevertheless, 902 participants completed the questionnaire. The questionnaire was distributed through social media platforms using the snowball sampling technique.

### 2.2. The Questionnaire Design

The survey was first written in the Arabic language and this linguistic version was validated by language specialists. Item development utilized the literature that was gathered from PubMed and Google scholar, and the objective was to develop items which could assess the knowledge about the risk factors for GUMs, misconceptions about these risk factors (as there are varying local beliefs regarding the causes of congenital defects, ranging from superstitions to myths), and attitudes towards preventative measures for GUMs. The format of questions was close ended and only completed questionnaires were submitted, in order to avoid the problem of missing data and to minimize information bias.

The draft of the survey began with 34 questions that included 10 items designed to assess knowledge, 10 items designed to evaluate misconceptions, and 14 items designed to measure attitudes. To satisfy the requirements of content validity, the LAWSHE technique was employed. The items were reviewed by 3 experts and after that, the content validity ratio (CVR) of each item was calculated. Items found to have CVRs lower than 0.99 were removed, which resulted in the exclusion of 3 questions from the knowledge construct (retaining 7 items), 2 from the misconceptions construct (retaining 8 items), and 3 from the attitudes construct (retaining 11 items). Therefore, the total items in the survey were 25 questions (Supplementary Material).

The first section focused on demographics (age, gender, marital status, educational level, socioeconomic status, and place of residence). The second section contained seven questions aiming to assess the participants’ depth of knowledge about the risk factors for GUMs or defects in children. The third section also included seven questions designed to assess the participants’ incorrect perceptions regarding the risk factors for developing GUMs. The fourth section contained questions to assess the participants’ attitudes towards GUM prevention. Bloom’s cut-off points were used to determine the overall outcome measures (to categorize the degree of knowledge, perceptions and attitudes) as follows:

1. Knowledge: Each knowledge question was posed with three alternative answers, namely, ‘Yes’ (3), ‘I don’t know’ (2), and ‘No’ (1), which totals up to 21 as the maximum possible score. Every respondent provided a score that was indicative of their level of knowledge, with highly scoring respondents providing correct answers to a greater number of the questions, which attracted more points at the coding stage. A cut-off score was deemed acceptable if at least 80% of participants scored 17 points or higher, or if between 60% and 80% scored 13 or more. Scores below 60% were classified as poor.
2. Perception (all questions were false beliefs): The possible scores were ‘Yes’, which scored 1 point, ‘I don’t know’, which scored 2 points, and ‘No’, which scored 3 points, so the overall highest possible score was 21. The total score determined the level of perception; the higher the total score the higher the level of perception, since high points were given for correct answers at the coding stage. The statistical thresholds for performance were defined as follows: a normal performance required a cut-off of over 80%, corresponding to a target score of approximately 17 marks or higher; a moderate performance was indicated by a cut-off of over 60%, equating to around 13 marks or more; and a low performance was characterised by a cut-off of less than 60%.
3. Attitudes: The participants responded to the seven items by choosing from a 5-point response scale, in which one was (1) ‘strongly disagree’ and five was (5) ‘strongly agree’—the maximum possible being 55. The total score simply showed the participants’ opinion; the higher the score the better the attitude, since the greater values were assigned when coding the responses. Positive attitudes were recorded for respondents scoring 80 percent (44 points and above), while a score of 60 percent (33 points and above) was regarded as neutral. For scores less than 60%, the respondents are said to have negative attitudes.

### 2.3. Construct Validity and Reliability of the Questionnaire

**Figure 1:**
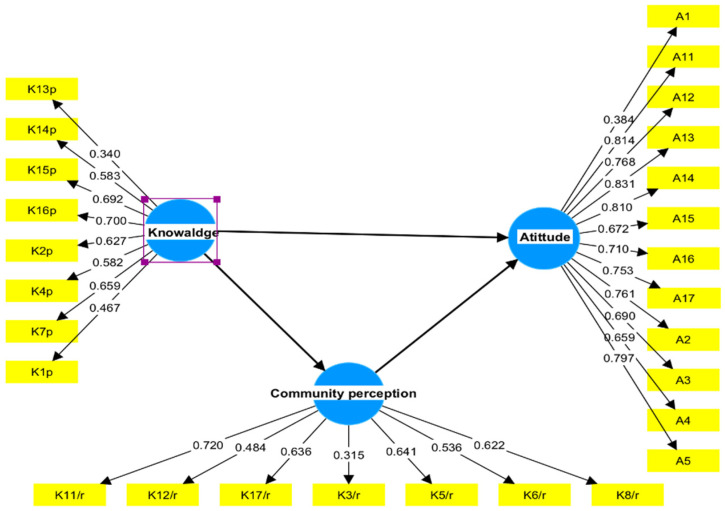
The measurement model and factor loadings for each questionnaire construct. Yellow rectangular shapes represent questions (factors, items), while blue circular shapes represent constructs (latent variables).

A pilot test was carried out with 40 participants to evaluate both the construct validity and reliability of the questionnaire and reliability using Smart PLS software version 4 (Smart PLS GmbH, Gewerbering 8, Tostedt, Germany, 2022). The partial least squares structural equation modeling (PLS-SEM) technique was used to estimate the factor loadings for each domain, to examine the validity (convergent and discernment) and reliability (internal consistency) of the questionnaire components. All components displayed composite reliability and a Cronbach’s alpha value greater than 0.7 when measured with the measurement model in Figure 1, indicating the questionnaire’s reliability (Supplementary File, Table S2). The average variance extracted (AVE) values of all constructs exceeded 0.5, showing convergent validity. The Fornell and Larker criteria were used to verify discriminant validity (Supplementary File, Table S3). Consistent PLS-SEM bootstrapping was used to test approximate model fit, yielding a standardized root mean square residual (SRMR) value of less than 0.1 (0.087; 95% confidence interval (CI): 0.056–0.190), indicating an acceptable model fit.

### 2.4. Statistical Analysis

The data were analyzed using SPSS version 25 (IBM Corp., Armonk, NY, USA). Frequencies and percentages were used to report categorical variables. Given that our primary outcome variables—knowledge, perceptions, and attitudes—are ordinal in nature, we employed ordinal logistic regression to explore their associations with demographic variables and to assess the impact of knowledge on perceptions and attitudes. Ordinal logistic regression is appropriate for modeling relationships involving an ordinal dependent variable, and allows us to account for the ordered nature of the data.

Potential confounders were selected based on the prior literature and statistical criteria. Variables such as age, gender, socioeconomic status, and educational level were considered, due to their known associations with both exposure and the outcomes. Variables that showed an association with the outcome at a significance level of *p* < 0.20 in univariate analyses were included in the multivariable model, to adjust for potential confounding effects.

The results are reported as crude odds ratios (CORs) in the univariate analysis and adjusted odds ratios (AORs) in the multivariate analysis, along with 95% confidence intervals (CIs). A *p*-value below 0.05 was considered statistically significant.

In addition, the Strengthening the Reporting of Observational Studies in Epidemiology (STROBE) Statement established the guidelines for reporting observational studies (Supplementary Materials Table S4).

## 3. Results

### 3.1. Demographics

A total of 902 respondents filled out a questionnaire assessing their knowledge regarding genitourinary malformations, and out of them the great majority were women (78.4%), and a large number were aged below twenty-five (73.8%). The majority were single (75.9%), graduated from university (75.4%), rated themselves as middle class (76%), and lived in metropolitan areas (41.9%). These social demographic data might have been crucial in determining the main conclusions. Since they were mainly young, educated women, this group might be exposed to more content about reproductive health, resulting in the better performance of this population in the reproductive health sector. Information seeking behaviour is common amongst women, which could also add to the factors that enable women to understand genitourinary diseases. Many of the participants aged 25 years or younger are university students or fresh graduates; therefore, this population is expected to be more open and likely have access to educational campaigns, as the levels of exposure to education increase the levels of exposure to health information. It can be inferred from the data that the majority of the participants were of middle-income backgrounds and may have poor chances to access advanced special health services and health education, except for the basic information. However, residing in metropolitan areas may mitigate these disadvantages to some extent compared to rural areas, as there are better facilities for health care, education, and health promotion activities in urban areas (Table 1).

### 3.2. Understanding of the Identified Risk Factors for the Development of GUMs

The majority of the participants were familiar with the risk factors for GUMs that had been pointed out in the references, such as consanguinity, exposure to radiation, maternal illness during pregnancy; however, few of them received a high general knowledge score, with most having a moderate score. From the logistic regression analyses, the odds of females and persons with above average social economic status possessing knowledge came out significantly greater, while no other demographic factors had significant relationships.

**Figure 2:**
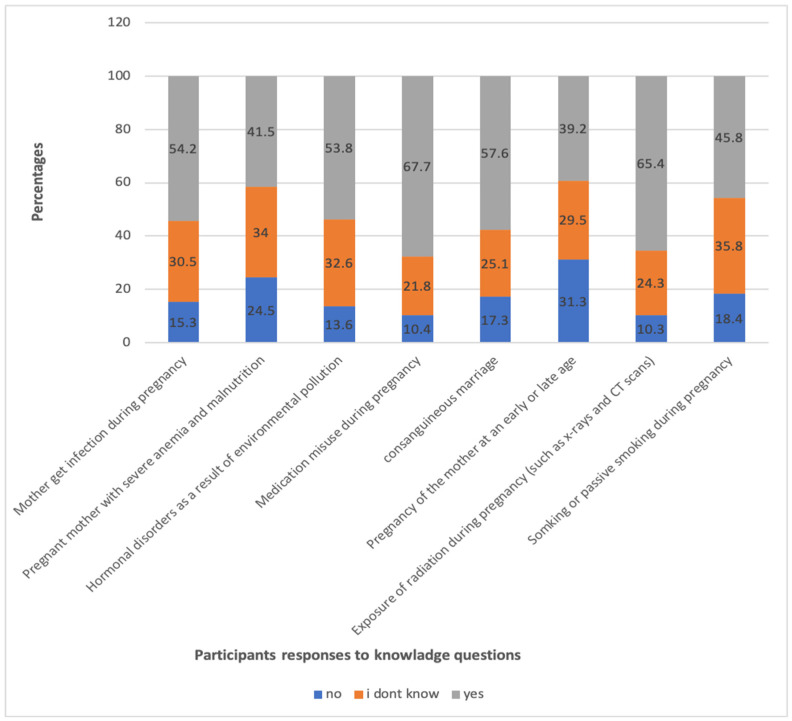
Presentation of the frequencies of respondents’ answers to the knowledge questions. These data are based on a sample size of 902 individuals who participated in the survey or study.

A total of 67% of participants knew that consanguineous marriage is a risk factor for GUM, 65% were aware that radiation exposure is also a risk factor for GUM, and 54% were aware that maternal illnesses during pregnancy are connected with GUM (Figure 2). However, only 11% received a strong overall knowledge score, while 62% received a moderate overall knowledge score (Table 2).

The relationship between demographic factors and knowledge level was explored using univariate and multivariable ordinal logistic regression. Females (COR 1.735; 95% CI 1.27–2.73) had significantly increased odds of achieving a higher degree of knowledge than males, according to univariate analysis. Females (COR 1.76; 95% CI 1.28–2.42) and above-average socioeconomic levels (AOR 2; 95% CI 1.02–3.96) were related to higher odds of having higher knowledge scores, but no other variables showed significant relationships (Table 3).

### 3.3. Incorrect Community Perception of Risk Factors for GUM Development

Interestingly enough, many respondents believed that conditions such as hormonal therapy during pregnancy, chronic maternal diseases, and conditions like pre-eclampsia increased the risk of GUM, while the odds of females achieving higher perception ratings as per univariate and multivariable analyses were significantly high.

**Figure 3:**
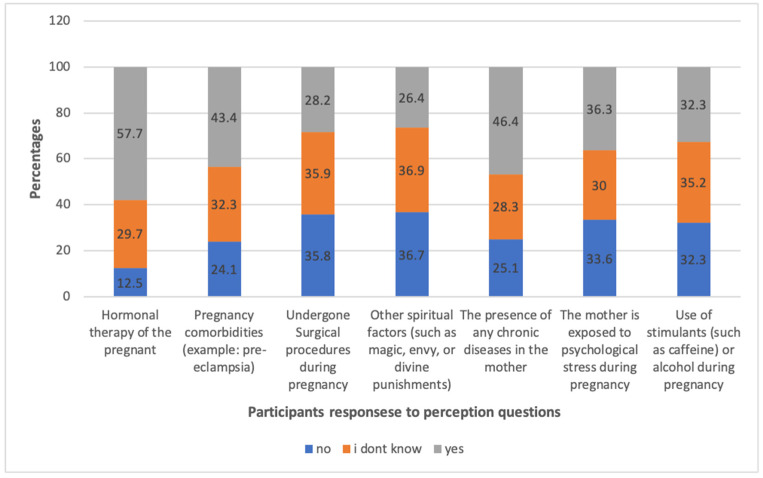
The distribution of frequencies corresponding to the answers provided by respondents to the questions addressing incorrect perceptions. The dataset comprises responses from a total of 902 participants.

A total of 57% of respondents believed that hormonal therapy during pregnancy increased the risk of GUM, 46% believed that the presence of any chronic disease in the mother increased the risk of GUM, and 43% believed that pregnancy-related diseases such as pre-eclampsia increased the risk of GUM (Figure 3). A total of 14% of respondents received a high overall perception score, while 58% had a moderate score (Table 2).

Females had higher odds of achieving higher perception ratings, according to univariate (COR 1.67; 95% CI 1.22–2.29) and multivariable analyses (AOR 1.6; 95% CI 1.2–2.28) (Table 4).

### 3.4. Attitude Towards Preventative Strategies for Genitourinary System Malformations

**Figure 4:**
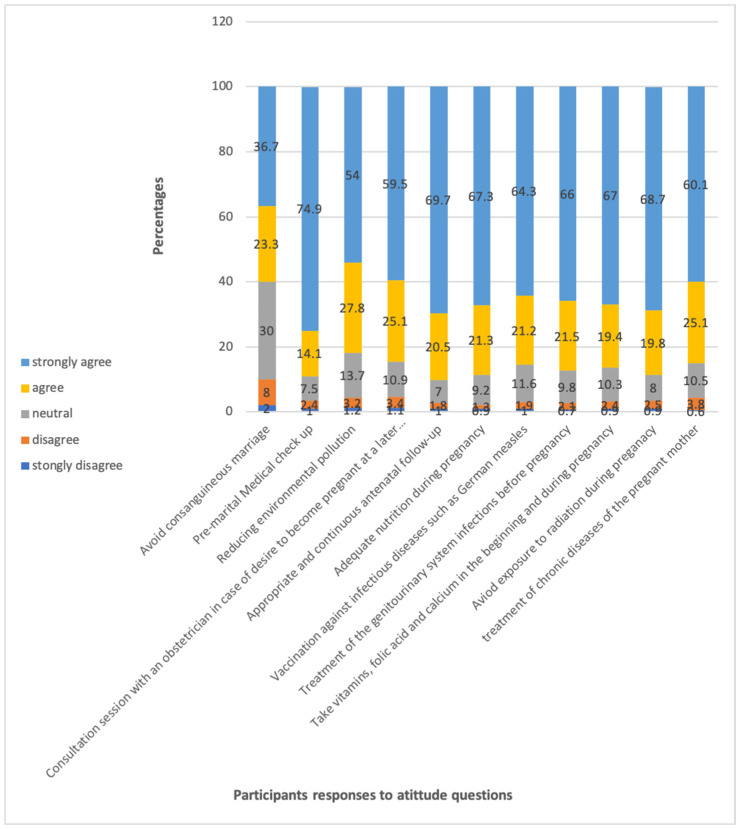
The distribution of respondents’ responses to the attitude questions, with a total sample size of 902.

A prevailing consensus indicated that adequate prenatal follow-ups, premarital medical examinations, and nutrition could facilitate enhancement. Most of the respondents had encouraging overall attitudes, while few were neutral. In both univariate and multivariable analysis, demographics were not found to be associated with attitude scores. A total of 74%, 69%, and 67%, respectively, strongly agreed on the need for proper and ongoing prenatal follow-ups, premarital medical check-ups, and optimal nutrition throughout pregnancy (Figure 4). A total of 83% of respondents had positive overall attitudes, with 13% having neutral attitudes (Table 2). In both univariate and multivariable analyses, there was no association between demographics and attitude scores (Table 5).

### 3.5. The Associations Between Knowledge, Perception and Attitude Scores

Univariate ordinal regression showed that the higher the knowledge scores, the higher the odds of scoring higher in attitudes. On the contrary, the higher the perception scores, the lower the attitude scores. In the multivariable analysis, favorable attitudes were positively predicted only by knowledge scores, and the prediction of incorrect attitudes was also significantly associated with higher levels of knowledge.

To assess the impact of the knowledge and perception scores on attitude scores, univariate ordinal regression revealed that a higher knowledge score (COR 13.5, 95% CI, 5 to 31) was associated with increased odds of having more positive attitude scores. However, higher perception scores (COR 0.24 95% CI, 0.1 to 0.5) were associated with lower odds of attitude scores. In multivariable analysis, only a high knowledge score (AOR 11.4 95% CI, 4.4 to 29) was found to be a significant predictor for positive attitude scores (Table 6). Ordinal regression also revealed that higher knowledge scores were significantly associated with lower incorrect perception scores (COR 0.024 95% CI, 0.11 to 0.47).

## 4. Discussion

Congenital genitourinary abnormalities present challenges to pediatricians and pediatric surgeons [12]. They comprise pathologies that are not well understood by parents or the general population [13]. This study explored people’s knowledge, perceptions, and attitudes towards these abnormalities. Despite the fact that the overall community knowledge showed quite reassuring results, more than a quarter of the participants expressed poor knowledge. Furthermore, the perception of risk factors appeared to be ambiguous. These findings may underscore the need to develop a strategy that involves the community, as well as physicians’ opinions, to deal with this problem.

Most of the study participants had moderate overall knowledge about GUMs and related risk factors. We also found that most participants had a positive attitude towards preventative strategies for GUMs. Most participants strongly agreed on the need for proper and ongoing prenatal follow-ups, premarital medical check-ups, and optimal nutrition throughout pregnancy. These findings suggest a strong awareness of the importance of these strategies, despite the lack of knowledge about the specific risk factors for GUMs.

In order to evaluate the representativeness of our sample, we compared our study’s demographics to those of Saudi social media users, as shown in the work conducted by Elkair et al. [14].

When we relate the sociocultural background of our respondents to that of the general Saudi public social media (SM) users for healthcare purposes, there are both similarities and differences. Similarly, the majority of participants (97%) are under the age of 36, which is consistent with the general demographic of Saudi SM users, the majority of participants (97%) are younger than 36 years. This means that the age of our study population generally matches the age range of the wider SM user population, which may suggest that the health-specific SM uses and preferences are somewhat similar. As has been the case among Saudi SM users, our study is also dominated by women, in fact, as much as 78.4% of our respondents were women. This conforms to the global reality of women being more involved in health-specific SM use. However, marital status appears to be altogether different; our sample has a significantly higher proportion of never married subjects (75.9%), while subjects from the general group of Saudi SM users tend to be married.

The educational level in our sample is similar to the general population’s, as 75.4% and 4% of the respondents possess a bachelor and master’s degree, respectively, which also explains the generally high educational level of SM users countrywide. Another element of comparison is economic status; a number of our respondents indicated an income level around the average, which aligns with the middle to upper income range (2000–10,000 SAR monthly) that most Saudi SM users possess. But our research, in addition to the average group, also definitely has smaller groups, those with below average income (4.4%) and above average income (19.4%). There is consistency as far as residency is concerned. In both scenarios urban areas are expected to prevail. In this study, 86.6% of the participants live in urban centers (including administrative cities, industrial cities, and governates) while only 13.4% are situated in rural areas (villages and small villages). This reflects the general geographical thrust of the bulk of Saudi SM user research, which is urban based.

In general, our findings suggest that with respect to age, gender, education level, and urban area of residence, there are similarities between our sample and that of Saudi SM users, but there are differences with regard to marital status.

Few participants had strong knowledge scores. This finding may have implications for prevention and treatment efforts. A similar study reported that most participants had inadequate knowledge regarding congenital malformations [15]. Another study revealed limited knowledge about the causes of congenital malformations [16]. Furthermore, one study indicated that mothers have moderate knowledge about birth defects [17]. Many factors could have influenced the results of the current study. Gender significantly impacted knowledge scores, with women exhibiting notably higher scores than men, potentially linked to the inherent concerns of mothers regarding their pregnancy and children’s health. This finding is consistent with the results of prior studies, which have found that women have a higher level of awareness than men as regards health issues [18,19,20]. However, marital status was not an indicator of higher knowledge scores in the current study, as most participating women were unmarried. This may require the need for targeted counseling and educational programs. Individuals with above-average socioeconomic status had higher odds of having high knowledge scores. This association was reported previously [17]. In contrast, average–below average participants showed lower knowledge scores, which could be due to limited exposure to contemporary educational resources. Thus, this should be taken into consideration while implementing educational strategies about GUMs, to overcome this observation. Most of the participants in our study were approximately 25 years old. Although knowledge was found to be significantly correlated with age in previous studies with similar age predominance [21,22], this correlation was not significant in ours. Positive associations between educational level and the knowledge of congenital anomalies and their risk factors have been previously documented [15,18,23], yet, we identified a non-significant relationship between level of education and the knowledge, attitudes, and perceptions of GUMs. Nevertheless, we found that more than half of the participants had satisfactory knowledge.

Previous studies have found that drugs, tobacco, radiation, environmental toxins, and maternal infections are crucial factors in the etiology of congenital fetal anomalies in general, and GUMs in particular [24,25]. Most of the current study respondents believed that these factors were important.

A considerable percentage of respondents believed that hormonal therapy during pregnancy increases the risk of GUMs, and some believed that the presence of any chronic disease in the mother increases the risk. This coincides with a prior study that showed similar incorrect perceptions and beliefs [15]. The noted misconceptions indicate the need for accurate health messaging in public education initiatives. Participants with higher knowledge scores were more likely to have a positive attitude towards preventative strategies for GUMs. Contrarily, higher perception scores were associated with lower odds of having positive attitude scores. This indicates that a high level of knowledge is a significant predictor of positive attitudes towards prevention. Therefore, increasing knowledge about GUMs could help improve attitudes towards preventative measures. Our findings are consistent with those of a previous study that assessed women’s knowledge about and attitude towards congenital anomaly risk factors in pregnancy, in which knowledge was significantly associated with a more positive attitude towards preventative strategies [26].

Furthermore, a high level of knowledge is significantly associated with low incorrect perception scores, indicating that knowledge is important in dispeling misconceptions about maternal and child health [20,21].

Overall, the findings of the current study highlight the importance of education and knowledge in promoting positive attitudes towards maternal and child health. Healthcare providers and policymakers should prioritize educational interventions that promote accurate knowledge and dispel misconceptions to improve attitudes as well as maternal and child health outcomes. However, we did not observe an association between demographic variables and attitude scores in either the univariate or multivariate analysis. This finding implies that the recognition of prenatal care and nutrition during pregnancy is important. It is consistent across different demographic groups. It may give a clue for understanding the significance of these factors universally, although it does not depend on factors such as age, gender, or other demographic characteristics. These results highlight the widespread agreement on the importance of proper prenatal follow-ups, premarital medical check-ups, and optimal nutrition throughout pregnancy. The absence of demographic associations further emphasizes the universal recognition of these factors. These findings contribute to the understanding of attitudes towards prenatal care and nutrition. They provide valuable insights for healthcare professionals and policymakers in promoting and implementing effective strategies to ensure the wellbeing of expectant mothers and their babies.

This study has certain limitations. The study’s cross-sectional design makes establishing causality between knowledge and attitudes impossible. The outcome might be affected by the questionnaire employed to assess the participants’ metric results of their knowledge about GUMs. Furthermore, the interpretation of some questions might vary depending on the participants’ conjuncture, while certain answer options may be interpretated differently among respondents, which could influence the results’ validity. Moreover, our findings suggest that with respect to age, gender, education level, and urban area of residence, there are similarities between our sample and that of Saudi SM users, but there are differences with regard to marital status, which might give rise to selection bias; therefore, our findings should be cautiously generalized.

## 5. Conclusions

According to our findings on the community knowledge, attitudes, and perceptions of GUMs, we conclude that the community has moderate overall knowledge regarding the etiology of and preventative measures against GUMs. However, a considerable percentage of the study population had a low level of knowledge, negative attitudes, and incorrect perceptions regarding these abnormalities. Therefore, this research indicates that public health campaigns should target specific misconceptions regarding GUM risk factors. Premarital and prenatal counseling programs incorporate information on GUM prevention to enhance community awareness. They also should be aimed at premarital and newly married couples, to reduce the incidence of GUMs. In addition, the widely adopted national premarital counseling program must address GUMs as a type of congenital malformation. Despite the limited number of participants, this study highlights the lack of knowledge about GUMs as a problem on the national level.

## Figures and Tables

**Table 1:** The frequencies of various demographic variables, with a total sample size (N) of 902 individuals.

Variables		Frequency	Percent
Age	≤25	666	73.8
	26–35	125	13.9
	36–50	89	9.9
	>50	22	2.4
Sex	Male	195	21.6
	Female	707	78.4
Marital status	Single	685	75.9
	Married	217	24.1
Education	Non-Educated	4	0.4
	Less than Bachelor	182	20.2
	Bachelor	680	75.4
	Postgraduate	36	4
Socioeconomic status	Below Average	40	4.4
	Average	687	76.2
	Above Average	175	19.4
Residency area	Administrative City	378	41.9
	Industrial City	51	5.7
	Governorate	352	39
	Village	119	13.2
	Small Village	2	0.2

**Table 2:** Classification of overall knowledge, incorrect perceptions, and attitude scores, with reference to a total sample size (N) of 902 individuals.

Domain	Level	Frequency	Percent
Knowledge about the factors that may lead to malformations or defects in the genitourinary system in children	Poor	232	25.7
	Moderate	563	62.4
	Good	107	11.9
Community perception about unexpected malformations or defects in the genitourinary system in children	High	132	14.6
	Moderate	522	57.8
	Low	248	27.5
Attitude towards genitourinary system malformation prevention measures	Negative	26	2.9
	Neutral	125	13.9
	Positive	751	83.3

**Table 3:** The relationship between demographic factors and knowledge using univariate and multivariable ordinal logistic regression.

Predictors	Univariate Analysis				Multivariable Analysis			
	COR	COR 95% Confidence Interval		*p*-Value	AOR	AOR 95% Confidence Interval		*p*-Value
		Lower	Higher			Lower	Higher	
**Age:**								
26–35–≤25	0.701	0.486	1.01	0.058	0.797	0.526	1.21	0.286
36–50–≤25	0.726	0.475	1.11	0.14	0.79	0.458	1.37	0.398
>50–≤25	0.643	0.277	1.5	0.301	0.817	0.322	2.09	0.67
**Gender:**								
Female—Male	1.73	1.27	2.37	<0.001 *	1.76	1.28	2.42	<0.001 *
**Marital:**								
Married—Single	0.755	0.562	1.01	0.062	0.858	0.572	1.29	0.46
**Education:**								
Less than Bachelor—Non-Educated	0.931	0.1143	6.33	0.941	0.97	0.116	6.76	0.976
Bachelor—Non-Educated	1.044	0.1298	6.99	0.964	1.022	0.124	6.99	0.983
Postgraduate—Non-Educated	0.784	0.0904	5.75	0.81	0.914	0.103	6.84	0.929
**Socioeconomic:**								
Average—Below Average	1.55	0.841	2.88	0.159	1.682	0.903	3.14	0.101
Above Average—Below Average	1.8	0.927	3.51	0.082	2.008	1.021	3.96	0.043 *

* Significant, COR = Crude Odd Ratio, AOR = Adjusted Odd Ratio (N = 209).

**Table 4:** The relationship between demographic factors and incorrect perception using univariate and multivariable ordinal logistic regression.

Predictors	Univariate Analysis				Multivariable Analysis			
	COR	COR 95% Confidence Interval		*p*-Value	AOR	AOR 95% Confidence Interval		*p*-Value
		Lower	Higher			Lower	Higher	
**Age:**								
26–35–≤25	0.781	0.537	1.13	0.193	0.826	0.538	1.27	0.381
36–50–≤25	0.834	0.537	1.29	0.419	0.769	0.438	1.34	0.357
>50–≤25	0.549	0.243	1.24	0.15	0.577	0.233	1.43	0.235
**Gender:**								
Female—Male	1.67	1.22	2.29	0.001 *	1.655	1.204	2.28	0.002 *
**Marital:**								
Married—Single	0.895	0.663	1.21	0.468	1.069	0.706	1.62	0.751
**Education:**								
Less than Bachelor—Non-Educated	1.61	0.28	9.33	0.591	1.649	0.281	9.7	0.579
Bachelor—Non-Educated	1.49	0.263	8.47	0.65	1.455	0.252	8.41	0.673
Postgraduate—Non-Educated	1	0.158	6.31	1	1.077	0.168	6.89	0.938
**Socioeconomic:**								
Average—Below Average	1.18	0.638	2.21	0.593	1.249	0.669	2.34	0.486
Above Average—Below Average	1.25	0.638	2.44	0.52	1.403	0.713	2.77	0.328

* Significant, COR = Crude Odd Ratio, AOR = Adjusted Odd Ratio (N = 209).

**Table 5:** The relationship between demographic factors and attitudes using univariate and multivariable ordinal logistic regression.

Predictors	Univariate Analysis				Multivariable Analysis			
	COR	COR 95% Confidence Interval		*p*-Value	AOR	AOR 95% Confidence Interval		*p*-Value
		Lower	Higher			Lower	Higher	
**Age:**								
26–35–≤25	0.947	0.521	1.85	0.865	1.394	0.6828	3.04	0.382
36–50–≤25	2.295	0.918	7.69	0.116	3.899	1.2676	14.95	0.027
>50–≤25	2.187	0.445	39.54	0.449	3.48	0.5768	67.74	0.259
**Gender:**								
Female—Male	1.35	0.792	2.23	0.253	1.431	0.8267	2.4	0.186
**Marital:**								
Married—Single	1.08	0.644	1.89	0.779	0.703	0.3604	1.43	0.316
**Education:**								
Less than Bachelor—Non-Educated	4.3	0.2001	39.2	0.23	6.605	0.2961	64.99	0.13
Bachelor—Non-Educated	4.76	0.2265	41.6	0.192	7.391	0.3389	69.63	0.103
Postgraduate—Non-Educated	1.94	0.086	19.6	0.599	2.199	0.0943	23.56	0.54
**Socioeconomic:**								
Average—Below Average	1.65	0.276	3.8	0.276	1.665	0.6063	3.89	0.274
Above Average—Below Average	2.58	0.079	7.26	0.079	2.799	0.8982	8.04	0.061

COR = Crude Odd Ratio, AOR = Adjusted Odd Ratio (N = 209).

**Table 6:** The impact of knowledge and incorrect perception scores on attitudes scores using univariate and multivariable ordinal logistic regression.

Predictors	Univariate Analysis				Multivariable Analysis			
	COR	COR 95% Confidence Interval		*p*-Value	AOR	AOR 95% Confidence Interval		*p*-Value
		Lower	Higher			Lower	Higher	
**Knowledge Level:**								
Moderate—Poor	2.17	1.05	4.25	0.028	1.943	0.881	4.13	0.09
Good—Poor	13.57	5.83	31.74	<0.001 *	11.483	4.454	29.98	<0.001 *
**Perception Level:**								
Moderate—Low	0.439	0.213	0.828	0.016	0.869	0.406	1.73	0.7
High—Low	0.244	0.111	0.51	<0.001 *	0.73	0.302	1.72	0.476

* Significant, COR = Crude Odd Ratio, AOR = Adjusted Odd Ratio (N = 209).

## Data Availability

The datasets used and analyzed during the current study are available from the corresponding author upon reasonable request.

## Supplementary Materials

The following supporting information can be downloaded at: https://www.mdpi.com/article/10.3390/healthcare12242558/s1. Table S1 displays the questions comprising the questionnaire assessing knowledge, perception, and attitudes. Table S2 shows the results of the Cronbach’s alpha and the Composite reliability which guages the internal consistancy of the survey constructs. Table S3 shows the Fornell and Larker criteria which was used to assess the discriminant validity of the constructs. Table S4: STROBE Statement—checklist of items that should be included in reports of cross-sectional studies.
