# Supplementary material for: Community Awareness and Perceptions of Genitourinary Malformations: A Cross-Sectional Survey Study
Source: Healthcare (Basel). 2024 Dec 19;12(24):2558. doi: 10.3390/healthcare12242558 (PMC11675782; doi:10.3390/healthcare12242558)
Supplement: Supplementary file 1 [file healthcare-12-02558-s001.zip › healthcare-3302233-supplementary.pdf]

Supplementary Materials:

Table S1 Displays the questions comprising the questionnaire assessing knowledge, perception, and attitudes.

No.	Questions	Answers				
-	*Age:*	Less than 25	26-35	36-50	Older than 50	
-	*Gender:*	Male	Female			
-	*Marital status:*	Single	Married			
-	*Education:*	Non-Educated	Less than Bachelor	Bachelor	Postgraduate	
-	*The standard of living:*	Below Average	Average	Above Average		
-	*Place of residence:*	Administrative City	Industrial City	Governorate	Village	Small Village
A	*Factors that may lead to abnormalities or defects in the genitourinary system in children:*					
1	The mother is exposed to an infection during pregnancy	Yes	No	I don't know		
2	Pregnant mother with severe anemia and malnutrition	Yes	No	I don't know		
3	Hormonal treatments for pregnant mothers	Yes	No	I don't know		
4	Hormonal imbalances due to environmental pollution	Yes	No	I don't know		
5	Diseases associated with pregnancy (example: preeclampsia)	Yes	No	I don't know		
6	Performing surgeries on the mother during pregnancy	Yes	No	I don't know		
7	Misuse of medications during pregnancy	Yes	No	I don't know		
8	Other spiritual factors (such as magic, envy, or divine punishments)	Yes	No	I don't know		
B	*Children suffering from genitourinary diseases or being born with malformations of the genitourinary system are associated with the following:*					
1	The presence of chronic diseases in the mother	Yes	No	I don't know		
2	The mother is exposed to psychological or nervous stress during pregnancy	Yes	No	I don't know		
3	Parents who are related (consanguineous marriage)	Yes	No	I don't know		
4	The mother becomes pregnant at an early or late age	Yes	No	I don't know		
5	Exposure of the pregnant mother to radiation (such as X-rays and CT scans)	Yes	No	I don't know		
6	The pregnant mother smoked (positive) or inhaled smoke (negative)	Yes	No	I don't know		
7	Use of stimulants (such as caffeine) or alcohol during pregnancy	Yes	No	I don't know		
C	*Methods of preventing birth defects in the reproductive and urinary system during pregnancy:*					

**1**	Avoid consanguineous marriage	Strongly Agree	Agree	Neutral	Disagree	Strongly Disagree
**2**	Medical examination of the spouses before marriage	Strongly Agree	Agree	Neutral	Disagree	Strongly Disagree
**3**	Reducing environmental pollution	Strongly Agree	Agree	Neutral	Disagree	Strongly Disagree
**4**	A consultatio n session with an obstetrician in the event of a desire to become pregnant at a late age (35 - 40 years old)	Strongly Agree	Agree	Neutral	Disagree	Strongly Disagree
**5**	Appropriate and continuous follow-up with the doctor before delivery	Strongly Agree	Agree	Neutral	Disagree	Strongly Disagree
**6**	Adequate nutrition during pregnancy	Strongly Agree	Agree	Neutral	Disagree	Strongly Disagree
D	*To reduce the chance of developing deformities and diseases in the genitourinary system in children, the pregnant mother should do the following:*					
**1**	Vaccination against infectious diseases such as German measles	Strongly Agree	Agree	Neutral	Disagree	Strongly Disagree
**2**	Treating reproductive system infections before pregnancy	Strongly Agree	Agree	Neutral	Disagree	Strongly Disagree
**3**	Take vitamins, folic acid and calcium in early and during pregnancy	Strongly Agree	Agree	Neutral	Disagree	Strongly Disagree
**4**	Avoid exposure to radiation during pregnancy	Strongly Agree	Agree	Neutral	Disagree	Strongly Disagree
**5**	Immediate treatment of chronic diseases of the pregnant mother	Strongly Agree	Agree	Neutral	Disagree	Strongly Disagree
**6**	Reducing exposure to nervous and psychological stress during pregnancy	Strongly Agree	Agree	Neutral	Disagree	Strongly Disagree

Table S2 : Shows the results of the Cronbach's alpha and the Composite reliability which guages the internal consistancy of the survey constructs.

	Cronbach's alpha	Composite reliability (rho_c)
**Knowaldge**	**0.72**	**0.804**
**Community perception**	**0.661**	**0.77**
**Atittude**	**0.923**	**0.935**

Table S3 shows the Fornell and Larker criteria which was used to assess the discriminant validity of the constructs.

	Atittude	Community perception	Knowaldge
**Atittude**	**0.755**		
**Community perception**	**0.195**	**0.579**	
**Knowaldge**	**0.297**	**0.592**	**0.615**

Table S4 STROBE Statement—Checklist of items that should be included in reports of ***cross-sectional studies***

	Item No	Recommendation	Page No
**Title and abstract**	1	(*a*) Indicate the study's design with a commonly used term in the title or the abstract	1
		(*b*) Provide in the abstract an informative and balanced summary of what was done and what was found	1
**Introduction**			
Background/rationale	2	Explain the scientific background and rationale for the investigation being reported	2
Objectives	3	State specific objectives, including any prespecified hypotheses	2
**Methods**			
Study design	4	Present key elements of study design early in the paper	3
Setting	5	Describe the setting, locations, and relevant dates, including periods of recruitment, exposure, follow-up, and data collection	3
Participants	6	(*a*) Give the eligibility criteria, and the sources and methods of selection of participants	3
Variables	7	Clearly define all outcomes, exposures, predictors, potential confounders, and effect modifiers. Give diagnostic criteria, if applicable	3
Data sources/ measurement	8*	For each variable of interest, give sources of data and details of methods of assessment (measurement). Describe comparability of assessment methods if there is more than one group	3-4
Bias	9	Describe any efforts to address potential sources of bias	4
Study size	10	Explain how the study size was arrived at	3
Quantitative variables	11	Explain how quantitative variables were handled in the analyses. If applicable, describe which groupings were chosen and why	3-4
Statistical methods	12	(*a*) Describe all statistical methods, including those used to control for confounding	4
		(*b*) Describe any methods used to examine subgroups and interactions	4
		(*c*) Explain how missing data were addressed	4
		(*d*) If applicable, describe analytical methods taking account of sampling strategy	4
		(*e*) Describe any sensitivity analyses	4
**Results**			
Participants	13*	(a) Report numbers of individuals at each stage of study—eg numbers potentially eligible, examined for eligibility, confirmed eligible, included in the study, completing follow-up, and analysed	5-6
		(b) Give reasons for non-participation at each stage	5-6
		(c) Consider use of a flow diagram	5-6

Descriptive data	14*	(a) Give characteristics of study participants (eg demographic, clinical, social) and information on exposures and potential confounders	5-6
		(b) Indicate number of participants with missing data for each variable of interest	5-6
Outcome data	15*	Report numbers of outcome events or summary measures	5-6
Main results	16	(*a*) Give unadjusted estimates and, if applicable, confounder-adjusted estimates and their precision (eg, 95% confidence interval). Make clear which confounders were adjusted for and why they were included	5-6
		(*b*) Report category boundaries when continuous variables were categorized	5-6
		(*c*) If relevant, consider translating estimates of relative risk into absolute risk for a meaningful time period	
Other analyses	17	Report other analyses done—eg analyses of subgroups and interactions, and sensitivity analyses	
**Discussion**			
Key results	18	Summarise key results with reference to study objectives	7-8
Limitations	19	Discuss limitations of the study, taking into account sources of potential bias or imprecision. Discuss both direction and magnitude of any potential bias	9
Interpretation	20	Give a cautious overall interpretation of results considering objectives, limitations, multiplicity of analyses, results from similar studies, and other relevant evidence	9
Generalisability	21	Discuss the generalisability (external validity) of the study results	9
**Other information**			
Funding	22	Give the source of funding and the role of the funders for the present study and, if applicable, for the original study on which the present article is based	10
